# Systematic Review and Network Meta-Analysis of Noninvasive Brain Stimulation on Dysphagia after Stroke

**DOI:** 10.1155/2021/3831472

**Published:** 2021-11-03

**Authors:** Lingling Li, Hailiang Huang, Yuqi Jia, Ying Yu, Zhiyao Liu, Xin Shi, Fangqi Wang

**Affiliations:** ^1^College of Rehabilitation Medicine, Shandong University of Traditional Chinese Medicine, Shandong 250355, China; ^2^Innovative Institute of Chinese Medicine and Pharmacy, Shandong University of Traditional Chinese Medicine, Shandong 250355, China

## Abstract

**Background:**

Dysphagia is a common sequelae after stroke. Noninvasive brain stimulation (NIBS) is a tool that has been used in the rehabilitation process to modify cortical excitability and improve dysphagia.

**Objective:**

To systematically evaluate the effect of NIBS on dysphagia after stroke and compare the effects of two different NIBS.

**Methods:**

Randomized controlled trials about the effect of NIBS on dysphagia after stroke were retrieved from databases of PubMed, Embase, Cochrane Library, Web of Science, CNKI, Wanfang Data, VIP, and CBM, from inception to June 2021. The quality of the trials was assessed, and the data were extracted according to the *Cochrane Handbook for Systematic Reviews of Interventions*. A statistical analysis was carried out using RevMan 5.3 and ADDIS 1.16.8. The effect size was evaluated by using the standardized mean difference (SMD) and a 95% confidence interval (CI).

**Results:**

Ultimately, 18 studies involving 738 patients were included. Meta-analysis showed that NIBS could improve the dysphagia outcome and severity scale (DOSS) score (standard mean difference (SMD) = 1.44, 95% CI 0.80 to 2.08, *P* < 0.05) and the water swallow test score (SMD = 6.23, 95% CI 5.44 to 7.03, *P* < 0.05). NIBS could reduce the standardized swallowing assessment (SSA) score (SMD = −1.04, 95% CI -1.50 to -0.58, *P* < 0.05), the penetration-aspiration scale (PAS) score (SMD = −0.85, 95% CI -1.33 to -0.36, *P* < 0.05), and the functional dysphagia scale score (SMD = −1.05, 95% CI -1.48 to -0.62, *P* < 0.05). Network meta-analysis showed that the best probabilistic ranking of the effects of two different NIBS on the DOSS score is rTMS (*P* = 0.52) > tDCS (*P* = 0.48), the best probabilistic ranking of the SSA score is rTMS (*P* = 0.72) > tDCS (*P* = 0.28), and the best probabilistic ranking of the PAS score is rTMS (*P* = 0.68) > tDCS (*P* = 0.32).

**Conclusion:**

Existing evidence showed that NIBS could improve swallowing dysfunction and reduce the occurrence of aspiration after stroke, and that rTMS is better than tDCS. Limited by the number of included studies, more large-sample, multicenter, double-blind, high-quality clinical randomized controlled trials are still needed in the future to further confirm the results of this research.

## 1. Introduction

Swallowing seems to be a simple action, but it actually involves the coordination of multiple muscle groups, the regulation of the cranial nerves, and the central nervous system [1]. Dysphagia refers to the damage to the structure or function of the lower jaw, lips, tongue, soft palate, throat, and esophagus, resulting in food or water that cannot be safely and effectively delivered from the mouth to the stomach [2], which are a common sequelae of stroke with incidence of 37%-78% [3]. If not treated in time, serious complications such as dehydration, malnutrition, and aspiration pneumonia can occur, leading to an increase in mortality and hospitalization [4]. In addition, studies [5] have also shown that 41% of patients with poststroke dysphagia said that they feel anxious or experience panic when eating, which shows that it has an adverse effect on the patients' self-esteem and psychological and social participation. Therefore, improving the swallowing function and reducing the occurrence of various complications in the process of swallowing rehabilitation are particularly necessary.

At present, traditional treatment methods for poststroke dysphagia include neuromuscular electrical stimulation, feeding training, cold stimulation, acupuncture, biofeedback therapy, balloon dilation, and adjustment of food shape and eating posture. However, patients have different degrees of rehabilitation, and the effect is limited. Noninvasive brain stimulation (NIBS) techniques adjust the excitability of the cerebral cortex through electric or magnetic fields and accelerate the induction of neuroplasticity. Due to its advantages of noninvasiveness, easy operation, painlessness, and few side effects, NIBS has been widely used in the rehabilitation treatment of various brain dysfunctions, and the clinical application prospects are very broad [6, 7]. Repeated transcranial magnetic stimulation (rTMS) and transcranial direct current stimulation (tDCS) are the two most common methods of NIBS [8], and these have recognized effects on poststroke dysphagia.

The theoretical basis of NIBS is a model that involves interaction inhibition between the two hemispheres combined with the plasticity of the central nervous system. Changes of cortical plasticity are mainly achieved through neural network reconstruction, including functional compensation of the remaining brain regions around the affected lesion and the unaffected hemisphere [9, 10]. Under normal physiological conditions, the two hemispheres of the human brain are in a state of mutual inhibition and balance. After stroke, the affected cortex is damaged, which weakens the inhibitory effect on the unaffected cerebral hemisphere, enhances the excitability of the unaffected cerebral hemisphere, and breaks the original balance and stability [11, 12]. Based on the above theory, NIBS can be used to reduce the excitability of the unaffected hemisphere and enhance that of the affected hemisphere. rTMS is slightly different from tDCS. It achieves the effect of excitation or inhibition through frequency selection; that is, high frequency is applied to enhance cortical excitability and facilitate local nerve cells, while low frequency reduces cortical excitability and inhibits the activity of local nerve cells [13]. However, tDCS achieves the effect of excitation or inhibition due to different electrode positions; that is, when the anode was placed in the affected hemisphere, the resting membrane potential of the neurons is depolarized, and the excitability of neurons at the stimulated site is enhanced. Cathodic stimulation causes hyperpolarization of the membrane potential of the neurons and reduces cortical excitability [14]. Therefore, patients with dysphagia can choose the appropriate stimulation site and intensity according to the cerebral cortex injury, so as to achieve the effect of exciting or inhibiting the cerebral cortex and then improve the swallowing function.

At present, some studies have explored the rehabilitation effect of NIBS on poststroke dysphagia. However, the sample size of a single study is small, and the inclusion criteria and research methods are different. Evidence-based research on the rehabilitation of poststroke dysphagia by NIBS is sparse, and there is no evidence to show the difference of effects between the two different NIBS. This is not conducive to the development of evidence-based clinical practice of NIBS in treating poststroke dysphagia. Therefore, this study will systematically evaluate the rehabilitation effects of NIBS on poststroke dysphagia through evidence-based medicine and compare the effects of two different NIBS, in order to provide some reference for the application of NIBS in clinical rehabilitation in the future.

## 2. Materials and Methods

### 2.1. Search Strategy

Randomized controlled trials about the effect of NIBS on dysphagia after stroke were retrieved by two researchers (Zhiyao Liu and Xin Shi) from databases of PubMed, Embase, Cochrane Library, Web of Science, CNKI, Wanfang Data, VIP, and CBM, from inception to June 2021. The databases can be searched flexibly according to the combination of medical MeSH terms and general terms. Taking the PubMed database as an example, the specific retrieval strategy is as follows: #1 “stroke”[MeSH] OR cerebrovascular accident OR apoplexy OR brain vascular accident OR cerebral vascular accident OR hemiplegia OR CVA, #2 “transcranial direct current stimulation”[Mesh] OR “transcranial magnetic stimulation”[Mesh] OR non-invasive brain stimulation OR noninvasive brain stimulation OR transcranial electrical stimulation OR rTMS OR tDCS; #3 “randomized controlled trial”[MeSH] OR random OR random allocation OR RCT, and #4 #1 and #2 and #3.

### 2.2. Inclusion Criteria

1. Population: patients with poststroke dysphagia
2. Intervention: NIBS such as tDCS and rTMS
3. Comparison: sham-NIBS
4. Outcome: primary outcomes: dysphagia outcome and severity scale (DOSS), standardized swallowing assessment (SSA), and penetration-aspiration scale (PAS). Secondary outcomes: functional dysphagia scale (FDS) and water swallow test (WST)
5. Study design: randomized controlled trial (RCT)

### 2.3. Exclusion Criteria

If the study meets the following criteria, it should be excluded: non-RCTs such as self-control, cohort studies, case-control studies, and cross-sectional studies; those combined with other therapeutic interventions; those that involve patients with severe aphasia or cognitive impairment; those that have baselines that are not comparable or do not report baseline conditions; those with poor design or improper statistical methods; those with incomplete data, original data, or full-text documents that cannot be obtained after contacting the author; those without corresponding outcomes; those where the diagnostic criteria and the intervention time are not clear; and case reports, protocols, conference abstracts, animal experiments and reviews, and so on.

### 2.4. Data Extraction

Two researchers (Zhiyao Liu and Xin Shi) independently read the studies, extracted the data, and cross-checked. If there were any disagreement, it was discussed with the third researcher (Fangqi Wang). The extracted data include the descriptive information of the literature (first author, the year of publication) and general information of the included cases (age, duration, sample size, intervention length, stimulations, outcomes, etc.).

### 2.5. Risk of Bias (Quality) Assessment

Two researchers (Zhiyao Liu and Xin Shi) used the physiotherapy evidence database (PEDro) scale [15] to evaluate the methodological quality of the included studies, which comprised 11 items. The first item was not graded, with a full score of 10. A score of 7 is high quality, 5-6 is medium quality, and 4 is low quality. The risk of bias in the included studies was assessed by two researchers according to the *Cochrane Handbook for Systematic Reviews of Interventions* [16]. If the assessment results were different, it was transferred to the third researcher for judgment. Assessment items included random sequence generation, allocation concealment, blind methods, data integrity, selective reporting, and other biases. The quality assessment of the included studies was performed according to 3 options: high risk, low risk, and unclear.

### 2.6. Statistical Analysis

Traditional meta-analysis was carried out by RevMan 5.3 software: (1) Heterogeneity test: if *P* ≥ 0.1 and *I*^2^ ≤ 50%, there was no significant heterogeneity among the studies, and the fixed effect model was used; if *P* < 0.1 and *I*^2^ > 50%, there was significant heterogeneity among the studies, and the random effect model was used. When heterogeneity is high, subgroup analysis and sensitivity analysis were used to explore its source. (2) Calculation of effects: the outcome indicators included in the study are all continuous variables; the standard mean difference (SMD) was used to represent the magnitude of the effect and calculated 95% confidence interval (CI).

R 4.0.5 software was used to draw a network diagram, and ADDIS 1.16.8 software was used for statistical analysis to compare the differences in efficacy of the two NIBS. Based on the Markov chain Monte Carlo algorithm, network meta-analysis and probability rankings were carried out by four chains and consistency models. The initial value was set to 0.5, the step size to 10, and the number of iterations to 50,000. The first 20,000 iterations were used for annealing to eliminate the influence of the initial value, and the last 30,000 iterations were used for sampling. The potential scale reduced factor (PSRF) was calculated by comparing the variance within and between chains to evaluate convergence. PSRF values close to 1 indicated good convergence, and the results of the consistency model analysis are more reliable.

## 3. Results

### 3.1. Study Selection

**Figure 1:**
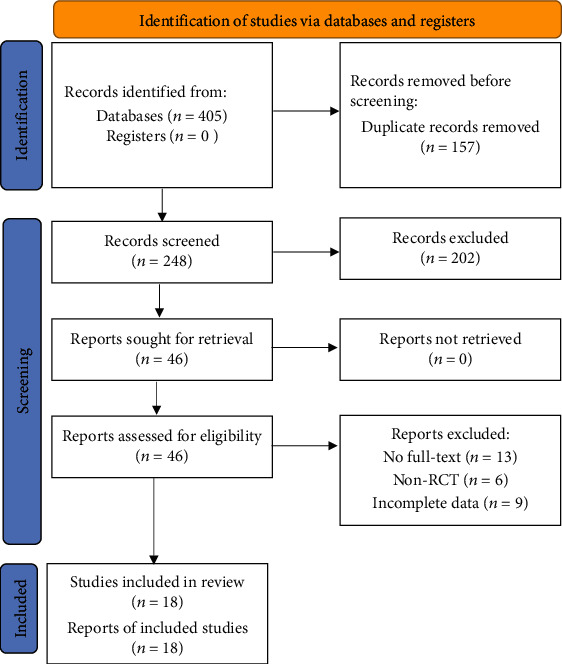
Screening process of literature selection.

A total of 405 studies were retrieved from the database; 248 studies were obtained after eliminating duplication with EndNote X9. After layer-by-layer screening, 18 studies [17–34] were finally included, with a total of 738 patients, 368 patients in the experimental group and 370 patients in the control group. The literature screening process is shown in Figure 1.

### 3.2. Study Characteristics and Risk of Bias (Quality) Assessment

**Figure 2:**
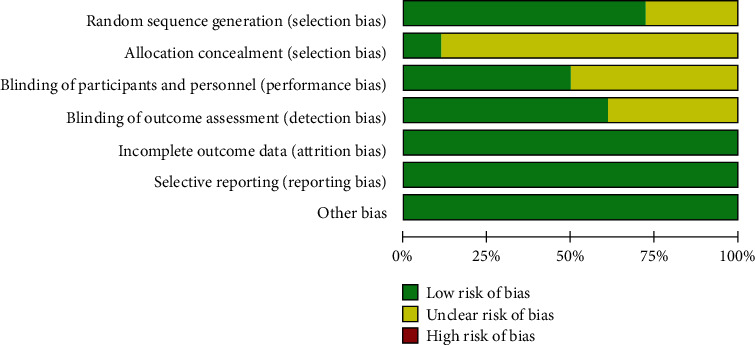
Bias risk assessment of included studies.

The basic information of the included studies is shown in Table 1. The quality evaluation results of the PEDro scores are shown in Table 2. Among these, there were 12 high-quality studies [17–20, 24–28, 30, 31, 34] and 6 medium-quality studies [21–23, 29, 32, 33] with an average score of 7.22. These 18 studies [17–34] mentioned random grouping, but 5 studies [18, 25, 27, 31, 34] did not mention specific random methods. Two studies [24, 34] have hidden the allocation schemes, while other studies have not described it. Nine studies [17–20, 24, 26, 28, 30, 31] blinded researchers or patients, and 11 studies [17–20, 24–28, 30, 34] blinded measurement results. The data contained in the studies is complete, and there was no selective report or other bias.  Figure 2 shows the bias risk assessment of the included studies.

### 3.3. Meta-Analysis

#### 3.3.1. DOSS

**Figure 3:**
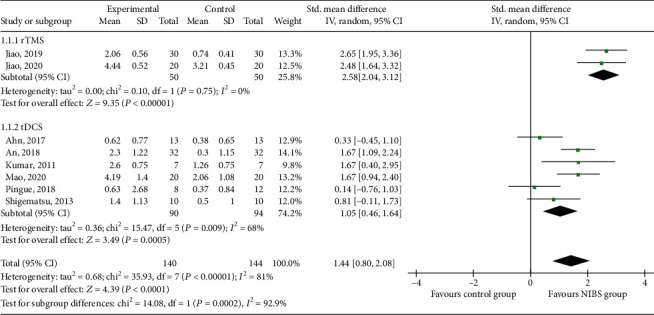
Effects of NIBS on DOSS scores of patients with dysphagia after stroke.

Eight RCTs [17–21, 23, 30, 31] reported DOSS scores, which involved 140 patients in the experimental group and 144 in the control group (Figure 3). Subgroup analysis showed that DOSS scores in the rTMS and tDCS groups were higher than those in the control group (SMD = 2.58, 95% CI 2.04 to 3.12, *P* < 0.05) and (SMD = 1.05, 95% CI 0.46 to 1.64, *P* < 0.05). Meta-analysis showed that the DOSS score in the NIBS group was higher than that in the control group (SMD = 1.44, 95% CI 0.80 to 2.08, *P* < 0.05).

#### 3.3.2. SSA

**Figure 4:**
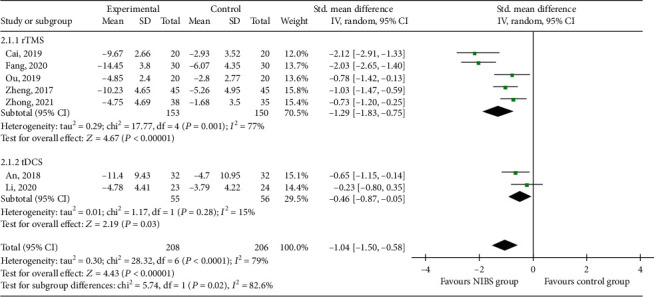
Effects of NIBS on SSA scores of patients with dysphagia after stroke.

Seven RCTs [21, 22, 26, 28, 29, 32, 34] reported SSA scores, which were from 208 patients in the experimental group and 206 in the control group (Figure 4). Subgroup analysis showed that SSA scores in the rTMS and tDCS groups were lower than those in the control group (SMD = −1.29, 95% CI -1.83 to -0.75, *P* < 0.05) and (SMD = −0.46, 95% CI -0.87 to -0.05, *P* < 0.05). Meta-analysis showed that the SSA score in the NIBS group was lower than that in the control group (SMD = −1.04, 95% CI -1.50 to -0.58, *P* < 0.05).

#### 3.3.3. PAS

**Figure 5:**
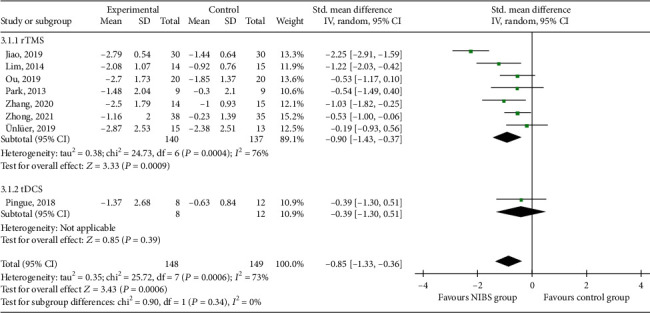
Effects of NIBS on PAS scores of patients with dysphagia after stroke.

Eight RCTs [20, 24, 25, 27, 29, 30, 33, 34] reported PAS scores, which were from 148 patients in the experimental group and 149 in the control group (Figure 5). Subgroup analysis showed that PAS scores in the rTMS group were lower than those in the control group (SMD = −0.90, 95% CI -1.43 to -0.37, *P* < 0.05). The PAS score in the tDCS group was not statistically significant compared with the control group (SMD = −0.39, 95% CI -1.30 to 0.51, *P* > 0.05), but most of the black rhombic blocks fell in the experimental group, with a tendency to lower PAS scores. Meta-analysis showed that the PAS score in the NIBS group was lower than that in the control group (SMD = −0.85, 95% CI -1.33 to -0.36, *P* < 0.05).

#### 3.3.4. FDS

**Figure 6:**
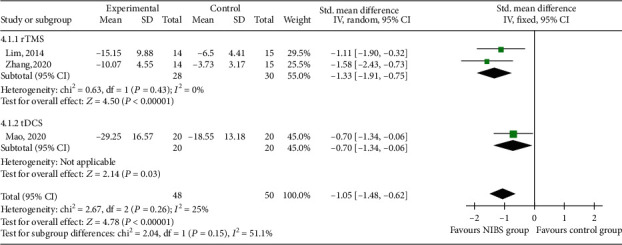
Effects of NIBS on FDS scores of patients with dysphagia after stroke.

Three RCTs [23, 25, 33] reported FDS scores, which were of 48 patients in the experimental group and 50 in the control group (Figure 6). Subgroup analysis showed that FDS scores in the rTMS and tDCS groups were lower than those in the control group (SMD = −1.33, 95% CI -1.91 to -0.75, *P* < 0.05) and (SMD = −0.70, 95% CI -1.34 to 0.06, *P* < 0.05). Meta-analysis showed that the FDS score in the NIBS group was lower than that in the control group (SMD = −1.05, 95% CI -1.48 to -0.62, *P* < 0.05).

#### 3.3.5. WST

**Figure 7:**
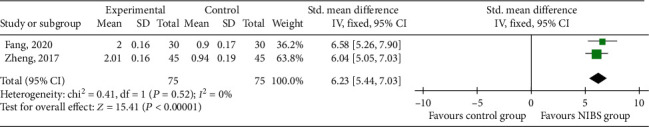
Effects of NIBS on WST scores of patients with dysphagia after stroke.

Two RCTs [26, 32] reported WST scores, which were of 75 patients in the experimental group and 75 in the control group (Figure 7). Meta-analysis showed that the WST score in the NIBS group was higher than that in the control group (SMD = 6.23, 95% CI 5.44 to 7.03, *P* < 0.05).

### 3.4. Network Meta-Analysis

#### 3.4.1. Evidence Network

**Figure 8:**
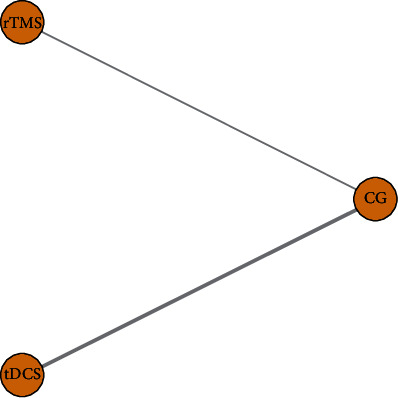
The network relationship of efficacy comparison of different NIBS.

Eighteen RCTs [17–34] were included in the analysis, eleven with rTMS intervention [24–34] and seven with tDCS intervention [17–23]. Taking the DOSS score as an example, the network relationship of efficacy comparison of different NIBS is shown in Figure 8. The connection between each ball represents the RCT, and there is a direct comparison between the two interventions. The thickness of the gray line represents the number of RCTs.

#### 3.4.2. Consistency Test

In this study, there was no closed loop between the interventions, so there was no need for a consistency test.

#### 3.4.3. Convergence Diagnosis

The primary outcomes of the eighteen RCTs [17–34], such as DOSS, SSA, and PAS, were analyzed by network meta-analysis, and the PSRF was 1.00, indicating satisfactory convergence.

#### 3.4.4. Probability Ranking

**Figure 9:**
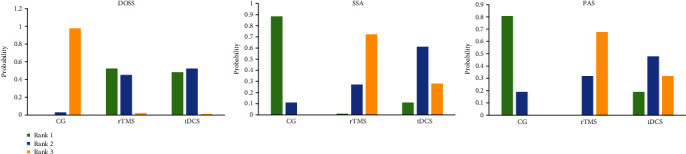
Probability ranking of outcomes of different NIBS.

The probability ranking of the network meta-analysis is shown in Table 3 and Figure 9. For the DOSS score, ranking 1 is best and ranking *N* is worst. For the SSA and PAS scores, ranking *N* is best and ranking 1 is worst.

Network meta-analysis showed that the best probabilistic ranking of the effects of two different NIBS on the DOSS score is rTMS (*P* = 0.52) > tDCS (*P* = 0.48), the best probabilistic ranking of the SSA score is rTMS (*P* = 0.72) > tDCS (*P* = 0.28), and the best probabilistic ranking of the PAS score is rTMS (*P* = 0.68) > tDCS (*P* = 0.32).

### 3.5. Adverse Reaction

Two studies [27, 34] reported dizziness, headache, or nosebleed after NIBS intervention, which was relieved quickly after rest. Adverse reactions were not reported in other studies.

### 3.6. Subgroup Analysis of Primary Outcomes

Subgroup analysis of DOSS, SSA, and PAS scores was performed according to duration, intervention length, and stimulation site. The results are shown in Table 4, which are basically consistent with the overall analysis results.

### 3.7. Sensitivity Analysis

The sensitivity analysis of the meta-analysis results was conducted by changing the effect model of each outcome and deleting one RCT each time. The results showed that there was no significant change compared with those before the analysis, indicating that the meta-analysis results were relatively robust.

### 3.8. Publication Bias

Taking the DOSS score as an example, the funnel plot analysis of the included studies is shown in Supplementary Materials. There is no obvious asymmetry in the inverted funnel plot, indicating that the results are reliable.

## 4. Discussion

The clinical value of NIBS, a new neuroregulation technology, to improve poststroke dysphagia is summarized here [35]. Our study systematically evaluated the rehabilitation effect of NIBS on poststroke dysphagia through evidence-based medicine and compared the effect differences between two NIBS.

Eighteen RCTs were included to evaluate swallowing dysphagia by primary outcomes of DOSS, SSA, and PAS. The DOSS scale [36] is divided into seven levels. The higher the level, the better the swallowing ability. The SSA scale [37] includes preliminary clinical examination, swallowing 5 ml of water three times and swallowing 60 ml of water. Meta-analysis showed that NIBS could improve the DOSS score and reduce the SSA score, with statistically significant results compared with the control groups (*P* < 0.05). Subgroup analysis showed that rTMS and tDCS were consistent with the results of the total combined effect in improving the DOSS score and reducing the SSA score, and both of them could improve the swallowing function of patients. The PAS scale [38] divides the degree of aspiration into eight grades, and the severity of assessment depends on the depth of food entering the airway and the ability to remove it. Grade 1 means normal, no food choking into the respiratory tract, and grade 8 means food choking into the respiratory tract, reaching below the glottis, and no effort was made to eliminate this reaction. The higher the rating was, the more serious the dysphagia. Meta-analysis showed that NIBS could reduce the PAS score and incidence of aspiration in patients with dysphagia compared with the control group, and this was statistically significant (*P* < 0.05). Subgroup analysis showed that rTMS and tDCS were not consistent with the total combined effect in reducing the PAS score. rTMS was effective while tDCS was ineffective. There was no strong evidence that tDCS could reduce the incidence of aspiration in dysphagia patients after stroke. The efficacy of tDCS in reducing the incidence of aspiration needs further research. As for the secondary outcomes of FDS and WST, this study showed that the NIBS group is superior to the control group.

In contrast to previous systematic reviews and meta-analyses [39, 40], the main outcomes of two different NIBS in improving the swallowing function of stroke patients were compared by network meta-analysis in our study, which provided some reference and basis for the selection of NIBS in clinical rehabilitation. Network meta-analysis shows that rTMS was superior to tDCS in improving swallowing function and reducing aspiration in stroke patients. rTMS continuously transmits multiple pulses with fixed frequency. rTMS using the induced current generated by magnetic field acting on the cerebral cortex changes the action potential of cortical neurons and affects the metabolism and nerve activity in the brain [41]. However, tDCS can only induce local currents in neurons, but cannot lead to spontaneous neuron discharges [42]. However, it has been reported that rTMS is at risk of causing epilepsy [43], while most of the adverse reactions of tDCS are transient dizziness and headaches [44]. In our study, adverse reactions such as dizziness, headache, or epistaxis were observed after rTMS intervention in the included studies of Ünlüer et al. [27] and Zhong et al. [34], while no significant adverse reactions were reported after tDCS intervention. Han et al.'s research [45] shows that the effect of cathode tDCS combined with low-frequency rTMS is better than single use. Therefore, the efficacy and safety of rTMS and tDCS in the treatment of poststroke dysphagia should be comprehensively considered, and the conclusions of our study are only for reference.

At present, the position of the swallowing center in the cerebral cortex is still unclear. Functional magnetic resonance imaging studies show that it may be related to the primary motor sensory cortex, insula, cingulate gyrus, prefrontal cortex, temporal lobe, and occipital region [46, 47]. Hamdy et al.'s research [48] shows that swallowing is innervated by bilateral nerves and is asymmetric. Bilateral cerebral hemispheres suppress balance and maintain normal swallowing function through the interaction of the corpus callosum. Hamdy et al. also believe that reorganization of the contralateral pharyngeal cortex is related to recovery of the swallowing function, which proves the role of complete hemisphere reorganization in the recovery of swallowing function after stroke [10]. After stroke, if the injury involves the cortical brainstem bundle, medulla oblongata reticular structure, or nerve nucleus, the swallowing muscles will not work normally, thus affecting the swallowing function [49]. The cortical medulla is a bridge connecting the brain stem and swallowing cortex. Michou et al.'s research [50] confirmed that the increase of cortical medulla excitability is related to the improvement of swallowing safety. There are relatively few studies on the mechanism of NIBS improving dysphagia after stroke, and there is no clear conclusion at present.

Although this study follows the criteria of systematic review and network meta-analysis report (PRISMA statement), there are certain potential biases. At present, there are relatively few RCTs about NIBS to improve dysphagia after stroke. The included studies are published in both Chinese and English, and the lack of relevant gray studies may lead to the bias of study selection. Some studies did not describe specific random methods, allocation concealment, or blind methods. Only seven studies reported follow-up, the follow-up time was short, and there was no long-term effect of NIBS on the swallowing function in stroke patients. The intervention time was 1~8 weeks, and the stimulation sites were unaffected, affected, or bilateral. The frequency and current of stimulation are also different. Our study could not determine the relationship between the length of the intervention period, the stimulation site and the intensity of stimulation, and the improvement of swallowing function.

## 5. Conclusions

To sum up, the existing evidence shows that NIBS can improve poststroke dysphagia and reduce the incidence of aspiration to a certain extent. rTMS is superior to tDCS, but it is necessary to carry out more large-sample, multicenter, double-blind, high-quality RCTs. In addition, NIBS has no obvious adverse reactions in the treatment of dysphagia after stroke, which is worthy of clinical application.

## Figures and Tables

**Table 1:** Characteristics of the included studies.

Studies	Country	Sample	Age	Duration (h/d/w/m)	Modality	Intervention length	Stimulation	Outcomes
Kumar et al., 2011 [17]	United States	7/7	79.71 ± 9.45/70.00 ± 11.07	80.29 ± 39.17/96.71 ± 42.52 (h)	tDCS	10 d	Pharyngeal motor cortex of the unaffected side, 2 mA	DOSS
Shigematsu et al., 2013 [18]	Japan	10/10	66.90 ± 6.30/64.70 ± 8.90	12.90 ± 7.80/12.10 ± 9.00 (w)	tDCS	10 d	Pharyngeal motor cortex of the affected side, 1 mA	DOSS
Ahn et al., 2017 [19]	Korea	13/13	61.62 ± 10.28/66.38 ± 10.67	12.27 ± 4.92/11.62 ± 4.56 (m)	tDCS	2 w	Bilaterally pharyngeal motor cortices, 1 mA	DOSS
Pingue et al., 2018 [20]	Italy	8/12	64.53 ± 18.54/67.77 ± 9.22	2 (m)	tDCS	10 d	Pharyngeal motor cortex of the affected side, 2 mA	DOSS, PAS
An, 2018 [21]	China	32/32	64.40 ± 12.20/66.70 ± 11.40	32.40 ± 13.40/31.80 ± 9.70 (h)	tDCS	2 w	Pharyngeal motor cortex of the unaffected side,2 mA	DOSS, SSA
Li et al., 2020 [22]	China	23/24	62.87 ± 8.71/63.38 ± 8.41	42.51 ± 61.63/37.6 ± 36.84 (d)	tDCS	3 w	Bilaterally pharyngeal motor cortices,1.4 mA	SSA
Mao et al., 2020 [23]	China	20/20	59.80 ± 7.27/61.25 ± 8.02	3.25 ± 2.24/3.60 ± 2.49 (m)	tDCS	8 w	Pharyngeal motor cortex of the unaffected side, 1.6 mA	DOSS, FDS
Park et al., 2013 [24]	Korea	9/9	73.70 ± 3.80/68.90 ± 9.30	59.90 ± 16.30/63.90 ± 26.80 (d)	rTMS	2 w	Pharyngeal motor cortex of the unaffected side, 5 Hz	PAS
Lim et al., 2014 [25]	Korea	14/15	59.80 ± 11.80/62.50 ± 8.20	30.30 ± 14.80/34.40 ± 10.10 (d)	rTMS	2 w	Pharyngeal motor cortex of the unaffected side, 1 Hz	PAS, FDS
Zheng et al., 2017 [26]	China	45/45	65.40 ± 7.80/66.50 ± 8.20	31.50 ± 10.80/32.50 ± 13.60 (d)	rTMS	20 d	From the anterolateral cortex of the skull to the front of the primary motor area, the lowest part of the anterior central gyrus, and the back part of the inferior frontal gyrus, 5 Hz	SSA, WST
Ünlüer et al., 2019[27]	Turkey	15/13	67.80 ± 11.88/69.31 ± 12.89	105.93 ± 49.02/101.38 ± 42.06 (d)	rTMS	1 w	Corresponding cortex area of suprahyoid muscle of the unaffected side, 1 Hz	PAS
Cai et al., 2019[28]	China	20/20	63.50 ± 10.90/61.10 ± 9.80	40.10 ± 7.80/46.90 ± 9.20 (d)	rTMS	2 w	Bilateral corresponding cortex area of suprahyoid muscle, 10 Hz	SSA
Ou et al., 2019[29]	China	20/20	64.10 ± 12.23/62.50 ± 13.27	10.00 ± 5.16/9.85 ± 5.54 (w)	rTMS	2 w	Corresponding cortex area of suprahyoid muscle of the unaffected side, 5 Hz	SSA, PAS
Jiao et al., 2019[30]	China	30/30	63.70 ± 4.70/65.20 ± 5.10	3.80 ± 1.40/4.30 ± 1.80 (w)	rTMS	4 w	From the anterolateral cortex of the skull to the front of the primary motor area, the lowest part of the anterior central gyrus, and the back part of the inferior frontal gyrus, 3 Hz	DOSS, PAS
Jiao et al., 2020[31]	China	20/20	62.90 ± 4.20/63.40 ± 4.60	3.80 ± 1.40/3.60 ± 1.10 (w)	rTMS	2 w	From the anterolateral cortex of the skull to the front of the primary motor area, the lowest part of the anterior central gyrus, and the back part of the inferior frontal gyrus of the affected side, 3 Hz	DOSS
Fang et al., 2020[32]	China	30/30	64.69 ± 8.41/65.12 ± 8.52	Not given	rTMS	2 w	Pharyngeal motor cortex of the unaffected side, 5 Hz	SSA, WST
Zhang et al., 2020[33]	China	14/15	55.21 ± 12.02/57.73 ± 15.78	11.36 ± 5.27/10.13 ± 4.17 (w)	rTMS	2 w	Corresponding cortex area of suprahyoid muscle of the unaffected side, 5 Hz	PAS, FDS
Zhong et al., 2021[34]	China	38/35	64.67 ± 10.87/62.34 ± 11.54	31.51 ± 35.52/23.22 ± 11.59 (d)	rTMS	2 w	Corresponding cortex area of suprahyoid muscle of the affected side, 5 Hz	SSA, PAS

**Table 2:** PEDro scores of the included studies.

Studies	1	2	3	4	5	6	7	8	9	10	11	Scores	Quality level
Kumar et al., 2011 [17]	Yes	1	0	1	1	0	1	1	1	1	1	8	High
Shigematsu et al., 2013 [18]	Yes	1	0	1	1	0	1	1	1	1	1	8	High
Ahn et al., 2017 [19]	Yes	1	0	1	1	0	1	1	1	1	1	8	High
Pingue et al., 2018 [20]	Yes	1	0	1	1	0	1	1	1	1	1	8	High
An, 2018 [21]	Yes	1	0	1	0	0	0	1	1	1	1	6	Medium
Li et al., 2020 [22]	Yes	1	0	1	0	0	0	1	1	1	1	6	Medium
Mao et al., 2020 [23]	Yes	1	0	1	0	0	0	1	1	1	1	6	Medium
Park et al., 2013 [24]	Yes	1	1	1	1	0	1	1	1	1	1	9	High
Lim et al., 2014 [25]	Yes	1	0	1	0	0	1	1	1	1	1	7	High
Zheng et al., 2017 [26]	Yes	1	0	1	1	0	1	1	1	1	1	8	High
Ünlüer et al., 2019 [27]	Yes	1	0	1	0	0	1	1	1	1	1	7	High
Cai et al., 2019 [28]	Yes	1	0	1	1	0	1	1	1	1	1	8	High
Ou et al., 2019 [29]	Yes	1	0	1	0	0	0	1	1	1	1	6	Medium
Jiao et al., 2019 [30]	Yes	1	0	1	1	0	1	1	1	1	1	8	High
Jiao et al., 2020 [31]	Yes	1	0	1	1	0	0	1	1	1	1	7	High
Fang et al., 2020 [32]	Yes	1	0	1	0	0	0	1	1	1	1	6	Medium
Zhang et al., 2020 [33]	Yes	1	0	1	0	0	0	1	1	1	1	6	Medium
Zhong et al., 2021 [34]	Yes	1	1	1	0	0	1	1	1	1	1	8	High

**Table 3:** Best probability ranking of different NIBS.

Interventions	DOSS	SSA	PAS
rTMS	0.52	0.72	0.68
tDCS	0.48	0.28	0.32
Control group	0	0	0

**Table 4:** Subgroup analysis of primary outcomes.

Subgroup analysis		Studies	SMD (95% CI)	*P*	*χ* ^2^	*I* ^2^ (%)	Tau^2^
DOSS							
Duration	≤2 w	2	1.67 [1.14, 2.19]	<0.0001	0	0%	0
	2 w to 6 m	5	1.67 [1.14, 2.19]	0.0008	25.60	84%	0.92
	≥6 m	1	0.33 [-0.45, 1.10]	0.41	—	—	—
Intervention length	≤2 w	6	1.18 [0.44, 1.91]	0.002	22.85	78%	0.64
	>2 w	2	2.17 [1.20, 3.13]	<0.0001	3.62	72%	0.35
Stimulation site	Unaffected side	3	1.67 [1.24, 2.09]	<0.0001	0	0%	0
	Affected side	3	1.15 [-0.25, 2.55]	0.11	14.92	87%	1.32
	Bilateral	2	1.50 [-0.79, 3.78]	0.2	18.97	95%	2.57

SSA							
Duration	≤2 w	1	-0.65 [-1.15, -0.14]	0.01	—	—	—
	2 w to 6 m	5	-0.93 [-1.42, -0.43]	0.0002	15.34	74%	0.23
	Not given	1	-2.03 [-2.65, -1.40]	<0.0001	—	—	—
Intervention length	≤2 w	5	-1.22 [-1.83, -0.61]	<0.0001	21.07	81%	0.38
	>2 w	2	-0.65 [-1.43, 0.14]	0.10	4.7	79%	0.25
Stimulation site	Unaffected side	3	-1.14 [-1.99, -0.29]	0.008	12.34	84%	0.47
	Affected side	1	-0.73 [-1.20, -0.25]	0.003	—	—	—
	Bilateral	3	-1.09 [-2.00, -0.17]	0.02	14.64	86%	0.56

PAS							
Duration	2 w to 6 m	8	-0.85 [-1.33, -0.36]	0.0006	25.72	73%	0.35
Intervention length	≤2 w	7	-0.61 [-0.87, -0.35]	<0.0001	4.99	0%	0
	>2 w	1	-2.25 [-2.91, -1.59]	<0.0001	—	—	—
Stimulation site	Unaffected side	5	-0.68 [-1.05, -0.32]	<0.0001	4.49	11%	0.02
	Affected side	2	-0.50 [-0.92, -0.09]	0.01	0.07	0%	0
	Bilateral	1	-2.25 [-2.91, -1.59]	<0.0001	—	—	—

## Data Availability

All research data used to support the findings of this study are included within the article and the supplementary materials.

## Supplementary Materials

Supplementary Materials Supplementary 1. S1: the funnel plot of the included studies.
