# Supplementary figures and images for: Systematic Review and Network Meta-Analysis of Noninvasive Brain Stimulation on Dysphagia after Stroke
Source: Neural Plast. 2021 Nov 3;2021:3831472. doi: 10.1155/2021/3831472 (PMC8580697; doi:10.1155/2021/3831472)

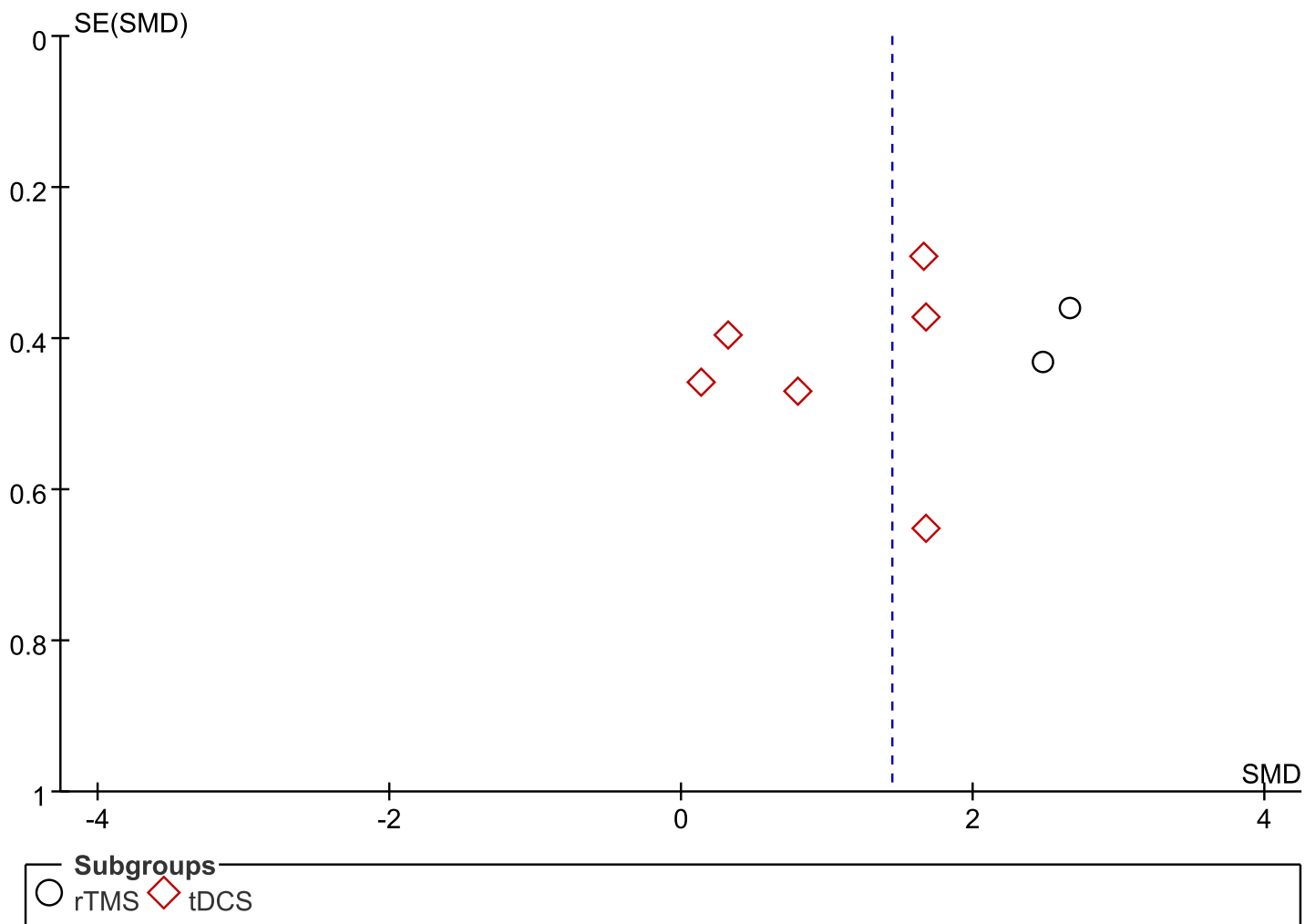

Supplement: Supplementary Materials — Supplementary 1. S1: the funnel plot of the included studies. [file 3831472.f1.zip › 3831472.f1/the funnel plot of the included studies.pdf]

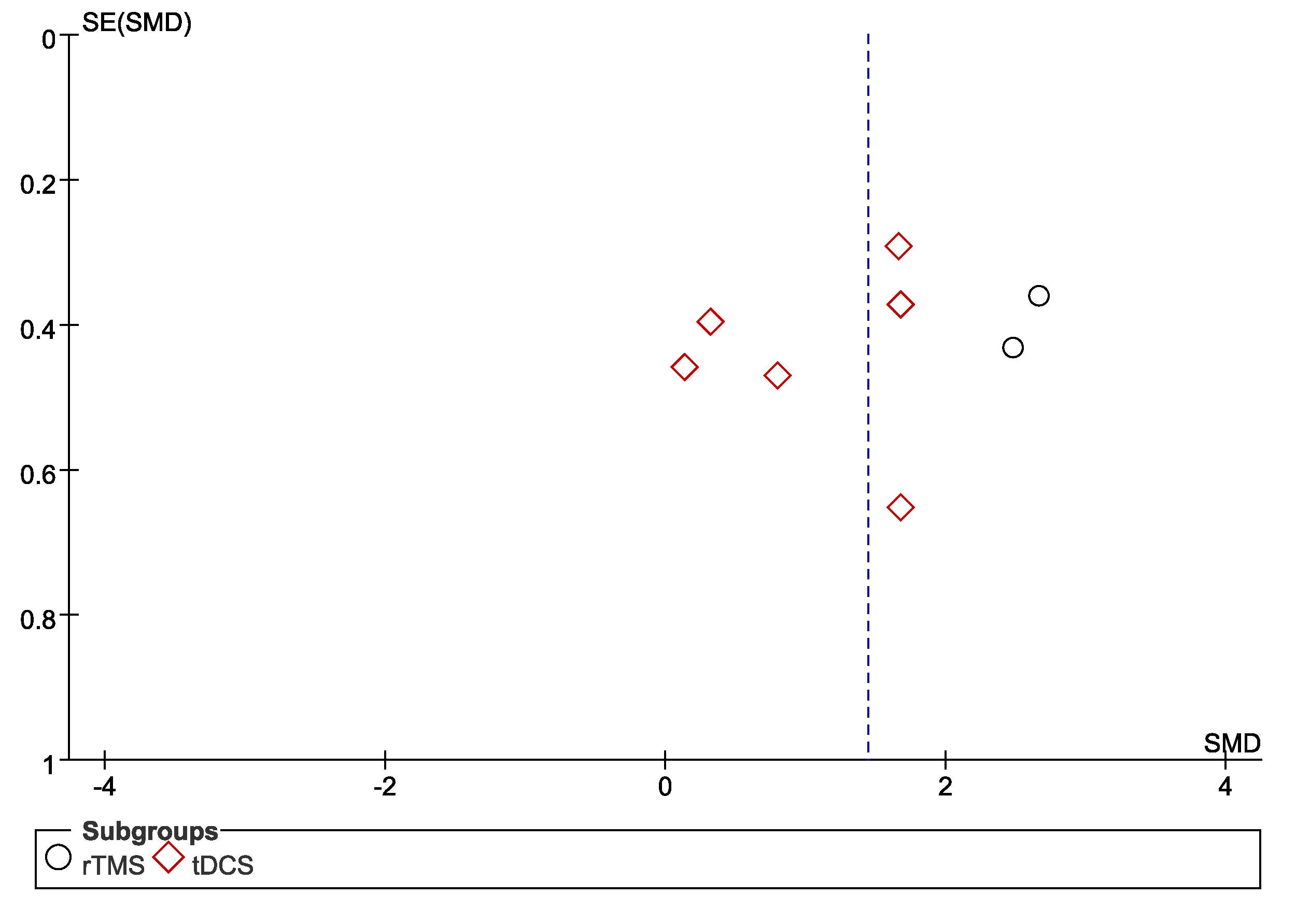

Supplement: Supplementary Materials — Supplementary 1. S1: the funnel plot of the included studies. [file 3831472.f1.zip › 3831472.f1/the funnel plot of the included studies.png]
